# Optimization of battery strengths in the Hodgkin-Huxley model

**DOI:** 10.1186/1471-2202-12-S1-P282

**Published:** 2011-07-18

**Authors:** Patrick Crotty, Thomas Sangrey

**Affiliations:** 1 Department of Physics and Astronomy, Colgate University, Hamilton, NY 13346, USA; 2 Department of Earth and Space Sciences, Columbus State University, Columbus, GA 31907, USA

## 

Simulations show that neurons are capable of operating over a much broader range of values of ionic reversal potentials than what is actually observed. Since the reversal potentials, which depend on the ionic concentration gradients across the membrane, have strong effects on the signaling properties and metabolic energy consumption rates of neurons, it is natural to hypothesize that the actual values are optimal for some functional property of the neuron involving energy and information, much as the ion channel densities and leak conductance appear to be [1,2]. Here we consider the Hodgkin-Huxley model of the squid giant axon and systematically investigate whether the reversal potentials for sodium and potassium, *E*_Na_ and *E*_K_, whose experimental values are respectively around +50 and -80 mV, minimize or maximize any simple combination of: (a) metabolic energy consumption during an action potential; (b) metabolic energy consumption during quiescence; (c) action potential velocity; (d) maximum firing frequency; and (e) the “energy efficiency” of the action potentials, which is the proportion of the metabolic energy associated with them that actually contributes to net inward or outward currents.

**Figure 1:**
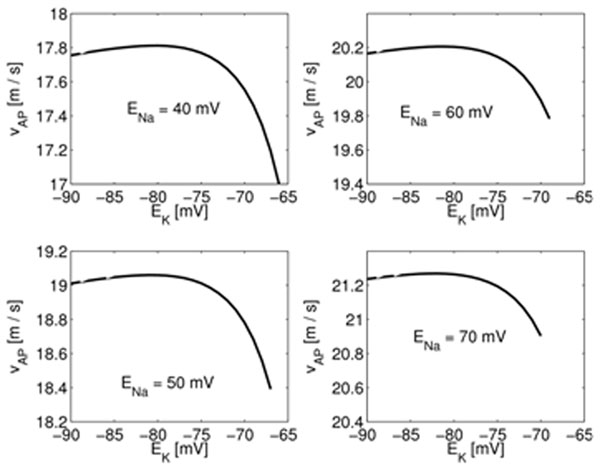
The velocity of action potentials (*v_AP_*) in the Hodgkin-Huxley model of the squid giant axon as a function of the potassium reversal potential (*E*_K_), with the sodium reversal potential (*E*_Na_) held fixed at various values. In all cases, *v_AP_* attains a maximum close to the experimental value of *E*_K_.

None of these quantities, nor any algebraically simple combination of them, has a maximum or minimum over the range of {*E*_Na_, *E*_K_} parameter space that we investigated. However, there are many functions for which the potassium reversal potential alone has an optimal value. Further analysis reveals that there are three basic underlying optimizations involving *E*_K_. The velocity of action potentials is maximized for a value of *E*_K_ in the experimental range (Fig. 1), and the product of the metabolic energy consumption during an action potential and at rest is minimized. The third optimization is for the ratio of action potential energy and maximum firing frequency, which is maximized for biological *E*_K_. The interpretation of this optimization may be clear only in the context of the larger squid escape-jet system.
